# Humidity sensor based on poly(lactic acid)/PANI–ZnO composite electrospun fibers

**DOI:** 10.1039/d1ra02842a

**Published:** 2021-08-26

**Authors:** Hemalatha Parangusan, Jolly Bhadra, Zubair Ahmad, Shoaib Mallick, Farid Touati, Noora Al-Thani

**Affiliations:** Qatar University Young Scientist Center (QUYSC), Qatar University P.O. Box 2713 Doha Qatar jollybhadra@qu.edu.qa; Centre for Advanced Materials (CAM), Qatar University P.O. Box 2713 Doha Qatar; Department of Electrical Engineering, College of Engineering, Qatar University P.O. Box 2713 Doha Qatar

## Abstract

The electrospinning technique has been successfully used to prepared micro-fibers of the poly(lactic acid)/polyaniline–zinc oxide (PLA/PANI–ZnO) composite. The polyaniline–zinc oxide (PANI–ZnO) nanocomposites are synthesized by hydrothermal and *in situ* polymerization methods. X-ray diffraction techniques are used to study the structural properties of the PLA/PANI–ZnO composite fibers and the PANI–ZnO nanocomposite. The average crystallite size of the PANI–ZnO nanocomposite is found to be 36 nm. The morphology and diameter of the composite fibers are analyzed by scanning electron microscopy (SEM). The average fiber diameter of the pure poly(lactic acid) (PLA) fiber is around 2.5 μm and that of the PLA/PANI–ZnO composite fiber is around 1.4 μm. Differential scanning calorimetry (DSC) provides the thermal properties of the PLA/PANI–ZnO composite fibers. The melting temperature (*T*_m_) for the pure PLA is observed at 149.3 °C, and it is shifted to 153.0 °C for the PLA/PANI–ZnO composite fibers. The enhanced thermal properties of the composite fibers are due to the interaction between the polymer and the nanoparticles. The water contact angle measurements probe the surface hydrophilicity of the PLA/PANI–ZnO composite fibers. The role of the PANI–ZnO nanocomposite on the sensing behavior of PLA fibers has also been investigated. The humidity sensing properties of the composite fiber based sensor are studied in the relative humidity (RH) range of 20–90% RH. The experimental results show that the composite fiber exhibited good response (85 s) and recovery (120 s) times. These results indicate that the one-dimensional (1D) fiber structure enhances the humidity sensing properties.

## Abstract 2

The electrospinning technique has been successfully used to prepared micro-fibers of the poly(lactic acid)/polyaniline–zinc oxide (PLA/PANI–ZnO) composite for humidity sensor application.

## Graphical abstract

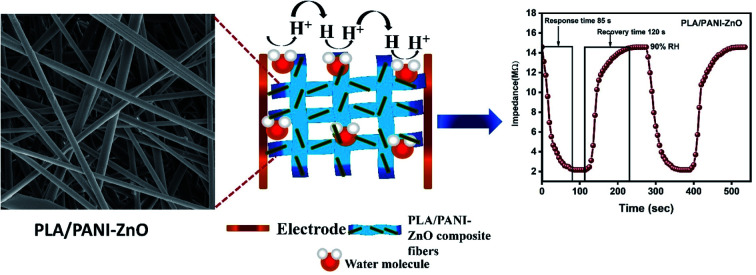

## 1. Introduction

The detection and monitoring of humidity are of great importance in many fields, such as textiles, agriculture, medicine, *etc.*^1^ An ideal humidity sensing material needs good sensitivity, a quick response, and recovery time. Different types of humidity sensing materials such as metal oxides, metal oxide semiconductors, ceramics, and polymers have attracted much interest in the humidity sensors field.^2,3^ Among the sensing materials, polymer nanocomposite based humidity sensors have gained much attention due to their high sensitivity, low cost, ease of fabrication, fast response and recovery times, and better stability.^4^ Venugopalan *et al.*^5^ investigated PVA based capacitive type sensors and Yao *et al.*^6^ investigated a gold–PVA encapsulated in gold particles based capacitive type humidity sensor. However, the problems encountered with PVA is that it is soluble in water and when used at higher humidity levels, the counter ions are exchangeable with H^+^ or OH^−^. Among the biodegradable polymers, polylactic acid is a biodegradable aliphatic polyester.^7^ PLA possesses excellent characteristics such as environmental stability, good biocompatibility, and unique mechanical properties, therefore, it has attracted interest as biomedical implants, and food packaging, and in the biomaterials industry. Co-polymerization, synthesis of polymer/metal oxide composite, and blending techniques extensively employed to improve the sensing properties of PLA. Among these techniques, polymer/metal oxide has gained considerable interest in research. The addition of a large surface area and small size of nanoparticles to the polymer matrix improves the mechanical and electrical properties and sensitivity of the polymer-based humidity sensor.^8^

The incorporation of TiO_2_ in PLA film has been previously reported by Shoaib *et al.*,^9^ and they have modified the PLA–TiO_2_ film surface by acetone etching, which showed superior morphological and sensing properties. Picciani *et al.*^10^ fabricated polyaniline/poly(lactic acid) [PLA/PANI] nanofibers based sensors by electrospinning technique, and they found that the composite fibers exhibited good sensitivity. Pure polyaniline (PANI) based material exhibit lower sensitivity than the PANI/metal oxide composite materials. For example, Singh *et al.*^11^ reported Fe_2_O_3_–PANI composite showed good sensitivity than the pure PANI. Polyaniline–zinc oxide (PANI–ZnO) based composite materials are also used as a sensor for humidity detection, and they found that the organic/inorganic (PANI–ZnO) based composite shows good sensitivity and quick response time.^12^ The hybrid nanofillers significantly enhance the sensing performance of a humidity sensor, rather than a single filler. Besides, 1D nanofibers, nanotubes, and nanowires have attracted considerable attention since they play an essential role in determining their chemical and physical properties.^13^ Electrospinning is one of the simple techniques for the synthesis of nanofibers. This method can be used to prepare different types of controlled and functionalized nanofiber. Therefore this method is the one of the most efficiently used method to fabricate the high-performance humidity sensors.^14^

The synergistic effect of a combination of two filler into the polymer matrix is a promising strategy to enhance the humidity sensing properties of polymer/metal oxide-based humidity sensors. In this work, we synthesized PANI–ZnO composite by using hydrothermal and *in situ*-polymerization method. The prepared composite fillers are embedded in the PLA matrix. The electrospinning technique is used for the fabrication of poly(lactic acid)/polyaniline–zinc oxide (PLA/PANI–ZnO) composite fibers. It is found that the PANI–ZnO nanoparticles influence the humidity sensing behavior of the samples.

## 2. Experimental

### 2.1. Materials

Aniline (C_6_H_5_NH_2_, *M*_w_ = 93.13), hydrochloric acid (HCl) and ammonium persulfate (APS) were obtained from Sigma Aldrich and were of high purity (99.9%) and used without any purification. Zinc acetate dehydrate (Zn(CH_3_COO)_2_·2H_2_O, *M*_w_ = 219.51), monoethanolamine (MEA) (C_2_H_7_NO, *M*_w_ = 61.08), and polyethylene glycol (PEG) were used for the synthesis of ZnO. Polylactic acid (PLA) is a high molar mass biopolymer (Ingeo™ Biopolymer 2003D) obtained from NatureWorks, LLC (USA), dichloromethane (DCM) with a density 133 kg L^−1^ and Folin–Ciocalteu reagent were obtained from BDH Middle East LLC, and dimethylformamide (DMF) (ACS, 99.8%) were purchased from the sigma Aldrich and used for the fabrication of PLA electrospun fibers. All reagents were used as received without further purification.

### 2.2. Synthesis of ZnO nanorods

For the preparation of ZnO nanorods, 2 g of zinc acetate dehydrated was dissolved in deionized water. The solution was stirred for one hour until a clear solution was obtained. Polyethylene glycol (PEG) was used as a surfactant. Monoethanolamine was added slowly to the above solution with constant stirring to get a homogeneous solution. The clear solution was transferred to the Teflon capped autoclave and kept at 140 °C for 3 h. The resulting precipitate was washed with deionized water and ethanol. Finally, dried at 80 °C in a hot air oven for 12 h and then calcined in a furnace at 400 °C for two hours.

### 2.3. Synthesis of PANI/ZnO composite

PANI/ZnO nanocomposite was prepared by the *in situ*-polymerization method. In a typical synthesis procedure, 0.2 M aniline and different weight percentage of 1 wt% of ZnO nanorods were dispersed into 50 mL of 0.01 M HCl solution, and the mixture was stirred in an ice-bath for 2 h 0.2 M of ammonium persulfate was added dropwise into the solution with continuous stirring for overnight, which acts as the oxidant. Then the precipitate powder was filtered and washed with distilled water and ethanol. The resulting precipitate was dried at 60 °C in a vacuum oven. Pure PANI was also synthesized following the same procedure without the addition of ZnO.

### 2.4. Synthesis of PLA/PANI–ZnO electrospun fibers

In order to prepare PLA/PANI–ZnO electrospun fibers, 12 wt% of PLA is dissolved in a mixed solution of 7 mL of dichloromethane (DCM) and 3 mL of dimethylformamide (DMF) and stirred at room temperature for six hours, respectively. Subsequently, 1 wt% of PANI, ZnO, and PANI–ZnO composites were dispersed in the DMF and DCM mixture (5 mL) using bath sonicator for 2 h and after that mixed with the PLA solution. The PLA nanocomposite solution was magnetically stirred overnight. The final PLA/PANI–ZnO composite solutions were loaded in a 10 mL syringe. The distance between the collector to the needle was 12 cm and at a flow rate of 1.5 mL h^−1^. The voltage of 12 kV was applied to the needle, and the nanofibers were collected on an aluminum substrate placed on the rotator.

### 2.5. Characterization techniques

The synthesized composite and its nanofibers structure are identified with an Empyrean, X-ray diffractometer within the 2*θ* range of 10° to 80° with Cu Kα1 radiation (1.5406 Å) operated at 45 kV and 40 mA. The morphology of the composites was examined by field emission scanning electron microscope (FESEM, Nova Nano SEM 450) and TEM (Philips CM12), respectively. Thermal analysis was done with 8500 PerkinElmer. The hydrophilic properties of the composites were measured by an OCA 35-Dataphysics, contact angle analyzer.

#### 2.5.1. Sensor fabrication

**Fig. 1:**
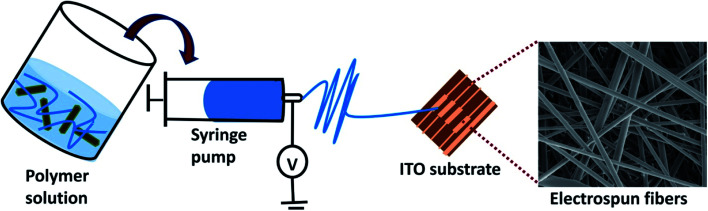
Schematic representation of sensor fabrication.

Fig. 1 presents the schematic representation of the PLA/PANI–ZnO based sensor. In this work, interdigitated ITO/glass substrates (from Ossila) of dimension 20 × 15 mm was used as the substrate. Before coating the sensing materials, the ITO/glass substrate is cleaned sequentially in acetone and deionized water in an ultrasonicator bath for 10 min. Then, the ITO surface was dried by nitrogen blow. To deposit the sensing film, the ITO substrate is placed on the aluminum foil. After spinning, the prepared films are dried in air at room temperature. The humidity sensing properties of PLA/PANI–ZnO fibers are measured using an assembled experimental setup.^9^ The humidity sensors were evaluated in a laboratory assembled humidity control chamber. To flow the humidity air and dry nitrogen inside the chamber an inlet and outlet valve were secure in the humidity chamber. Philips Respironics humidifier was used as a source of humidity air to increase the humidity level inside the chamber. While decreasing the relative humidity level, dry air was allowed to enter inside the chamber through the inlet valve, whereas humid air was evicted out from an outlet valve. MS5308 LCR meter was used to measure the resistance and capacitance of the sensor over a wide range of humidity levels. RS-6109 humidity meter was used to monitor the humidity level inside the chamber. All measurements were taken at room temperature.

## 3. Results and discussion

### 3.1. Structural and morphological properties of PANI–ZnO composite

**Fig. 2:**
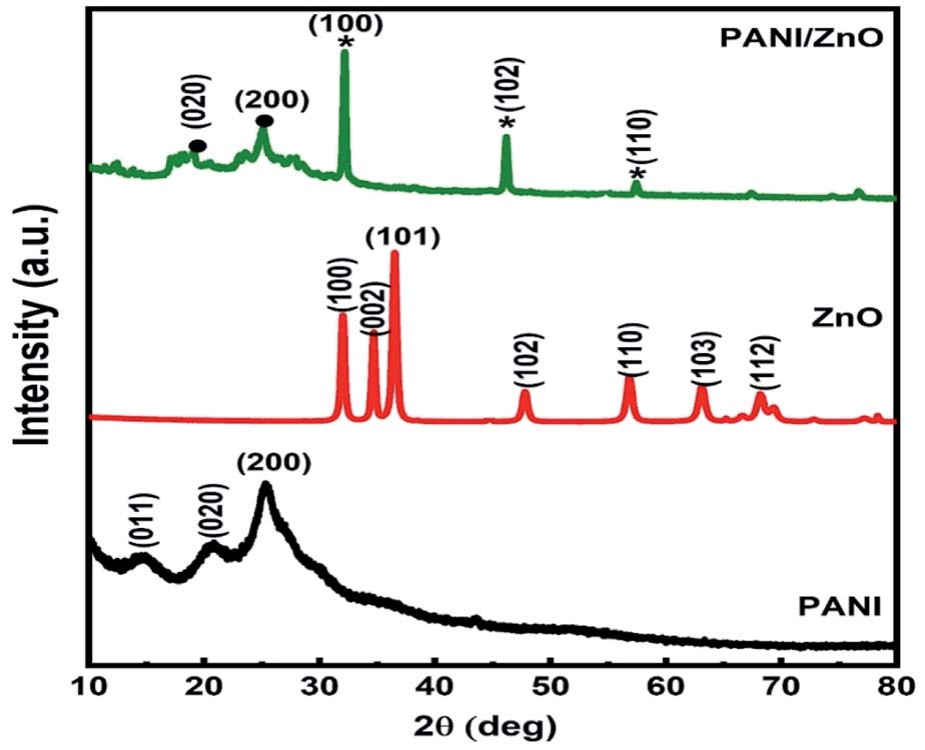
XRD pattern of PANI, ZnO, and PANI/ZnO nanocomposite.

The X-ray diffraction patterns of pure PANI, ZnO, and PANI–ZnO nanocomposites are investigated using the XRD spectra shown in Fig. 2. The XRD pattern of pure PANI exhibits the diffraction peaks 2*θ* = 15.23°, 20.77° and 25.39° corresponding to the (011) (020) and (200) planes of the pure PANI. The X-ray diffraction pattern of ZnO nanorods observed at 2*θ* 31.6°, 34.3°, 36.1°, 47.4°, 56.4°, and 62.7° corresponds to the crystal planes of (100), (002), (101), (102), (110), (103) and (112) respectively of the hexagonal phase of ZnO and are in good agreement with the JCPDS (79-0208).^15^ In the composite, both the diffraction peaks of PANI and ZnO are observed, but some of the ZnO peaks are reduced due to the deposition of the PANI layer on the ZnO surface. Also, the diffraction peaks of the ZnO planes at 31.6°, 47.4°, and 56.4° shifted towards the lower angle this confirms that the formation of PANI–ZnO composite structure.^16^ The crystallite size of the PANI–ZnO nanocomposite is estimated using Debye Scherrer's formula.1
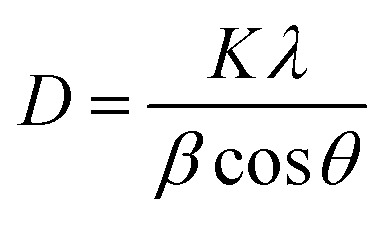
where *D* is the crystallite size, *β* is the FWHM, and *θ* is Bragg's angle. The average crystallite size of the PANI–ZnO nanocomposite was found to be 36 nm.

**Fig. 3:**
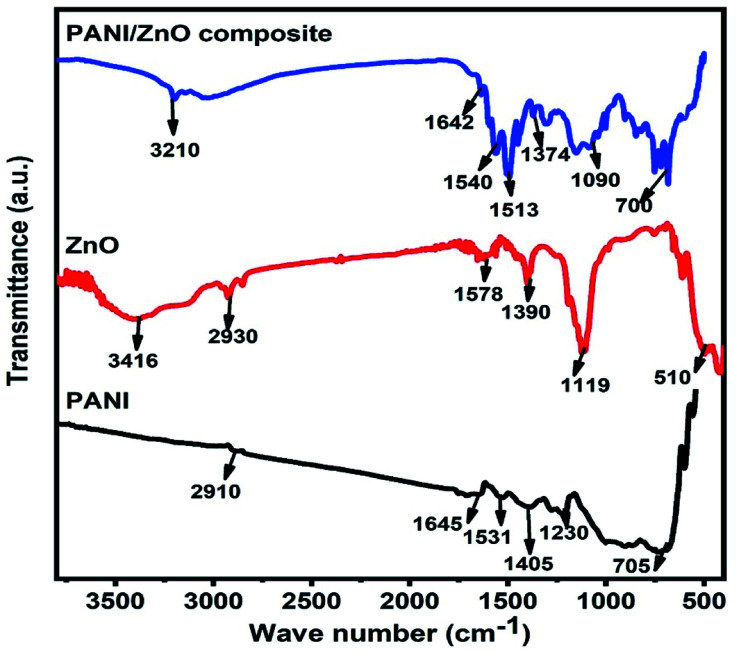
FTIR spectra of PANI, ZnO, and PANI/ZnO nanocomposite.

The structural properties of the pure PANI, ZnO and PANI–ZnO nanocomposite are studied by the FTIR spectroscopy. Fig. 3, shows the FTIR spectra of pure PANI, ZnO and PANI–ZnO nanocomposites. For pure PANI, the characteristics peaks observed at 2910 cm^−1^ and 1645 cm^−1^ are associated with the C–H and C=C stretching, respectively. The peaks at 1531 cm^−1^ and 1405 cm^−1^ are assigned to the aromatic ring stretching of the C=C–C bonds. These peaks are also called as the stretching vibrations of N–B–N and N=Q=N (B = benzoid, *Q* = quinoid).^17^ The characteristic peak at 1230 cm^−1^ is due to the C–N stretching vibration. The peak observed at 705 cm^−1^ corresponds to the NH_2_ wagging.^18^ For pure ZnO, the characteristics peak observed at 3416 cm^−1^ corresponds to the O–H stretching vibrations of water molecules. The vibration peak 2930 cm^−1^ indicating C–H stretching of alkane groups and 1578 cm^−1^ suggest the C=O bond. The peak observed at 1390 cm^−1^ is assigned to the vibration of –CH_2_. The vibrational band is observed at 510 cm^−1^ attributed to the Zn–O stretching vibration.^19^ Both PANI and ZnO peaks are observed in the PANI–ZnO nanocomposite and also the peaks are shifted towards the lower wave number. This may be due to the composite formation between PANI and ZnO.

**Fig. 4:**
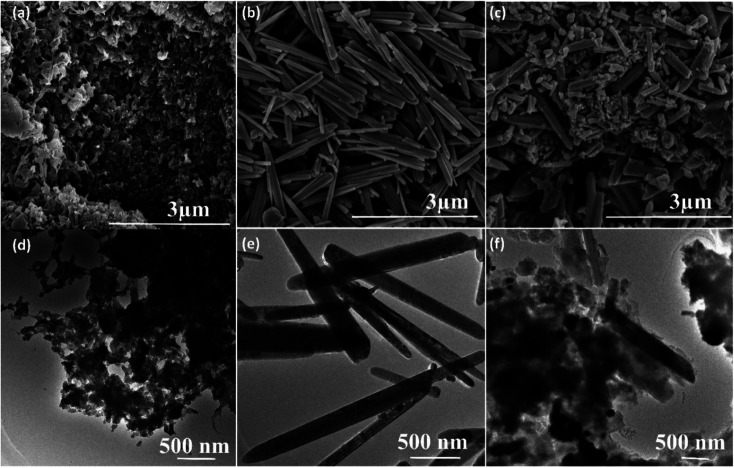
SEM images of (a) pure PANI, (b) pure ZnO, (c) PANI/ZnO nanocomposite, TEM images of (d) pure PANI, (e) pure ZnO and (f) PANI/ZnO nanocomposite.

The surface morphology of the pure PANI, ZnO and its nanocomposites are investigated by scanning electron microscopy (SEM). The SEM image of pure PANI in Fig. 4a reveals that a large number of agglomerated nanoparticles. Fig. 4b showed SEM images of pure ZnO; it elucidates the rod-like morphology. The average diameter of the pure ZnO rods (Fig. 4b) is found to be 120 nm to 150 nm. Fig. 4c showed the SEM images of PANI–ZnO composite, the combination of rods and agglomerated nanoparticles are observed in the composite sample. The TEM also used to analyzes the morphologies of pure PANI, ZnO, and its composites, and the images are shown in Fig. 4d–f. The TEM images of the PANI sample is displayed in Fig. 4d, which demonstrates that the pure PANI has shown agglomerated nanoparticles. Fig. 4e shows the transmission electron microscopy (TEM) images of the ZnO sample that reveals rod-like morphology, and the average diameter of the rod is 120–170 nm. The TEM images of PANI–ZnO composites support the observation of mixed morphologies of rods and agglomerated nanoparticle structures, as shown in Fig. 4e and f.

**Fig. 5:**
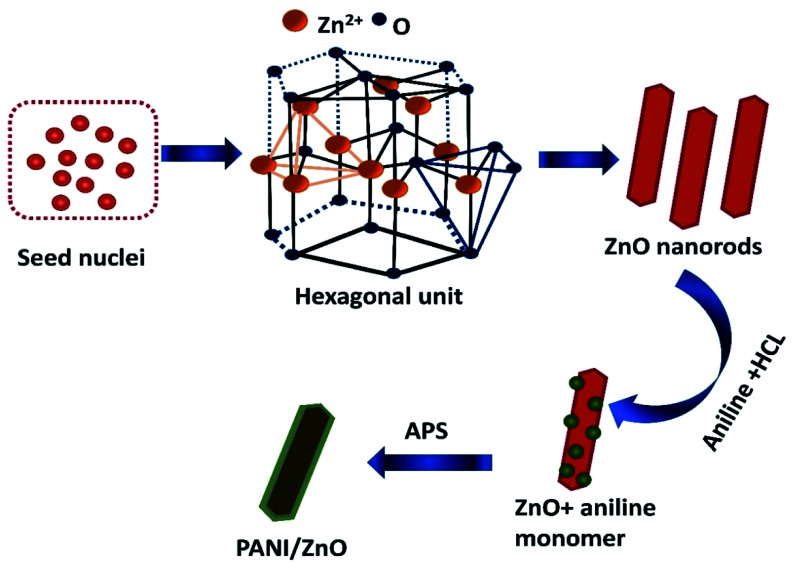
Schematic representation of polyaniline decorated ZnO rod.

The formation and growth mechanism of ZnO rods and polyaniline coated ZnO rods structures is shown in the schematic diagram in Fig. 5. In the beginning, the mixing of zinc acetate and monoethanolamine favorably react in aqueous solution to form a lot of zinc hydroxide seed nuclei. This decomposes under the hydrothermal treatment at a temperature of 140 °C to form ZnO nuclei. The formed zinc hydroxide seed nuclei undergo antistrophic growth along the preferential direction with the help of C_2_H_7_NO and OH^−^ leads to the one dimensional (1D) behavior of hexagonal crystal structure. The crystal nuclei are further developed into longer rods. This kind of large particle constitution can be described by the Ostwald ripening mechanism.^20^ For the preparation of PANI–ZnO nanocomposite, the prepared rods are dispersed in an aniline and HCl solution. After the addition of APS into an aniline solution, it begins to polymerize then PANI coats on the ZnO surface due to π–π* stacking interactions between the functional group and monomer of the aniline.^21^

### 3.2. Structural and thermal properties of PLA/PANI–ZnO composite fibers

**Fig. 6:**
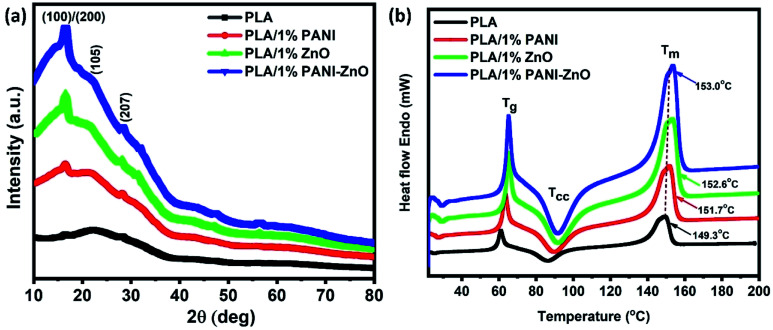
(a) X-ray diffraction patterns of PLA and its nanocomposite fibers, (b) thermal analysis of PLA and its composite fibers.

Fig. 6a shows the XRD patterns of PLA and its composites. The diffraction peaks of pure PLA observed at 2*θ* of 16.8° corresponds to (110) or (200) planes and the diffraction peaks at 22.4° and 27.5° corresponding to the crystalline planes of (105) and (207), respectively.^22^ The similar XRD patterns are observed in PLA/PANI, PLA/ZnO, and PLA/PANI–ZnO composites indicate that the addition of nanofillers does not affect the PLA crystal structure. It addition, the diffraction peaks correspond to the nanofillers are also observed in the range of 30° to 60° for the PLA nanocomposite fibers. The thermal properties of PLA and its composite fibers are studied by DSC analysis. The melting temperature (*T*_m_) of PLA fibers and PLA nanocomposite fibers are shown in Fig. 6b. It is observed that the addition of nanofillers increases the thermal properties of nanocomposite fibers. For neat PLA, the melting temperature is found at 149.3 °C, and for the composite, it is shifted to 151.7 °C, 152.6 °C and 153.0 °C. The slight increase in *T*_m_ for the composite is due to the incorporation of nanoparticles in the PLA matrix. This enhancement in the temperature values is attributed to the nucleating action of the fillers within the polymer.^23,24^ The insertion of nanoparticles in the PLA matrix can also influence the nucleation and polymer chain mobility and thus crystallization process.^25^

### 3.3. Morphological properties of PLA/PANI–ZnO composite fibers

**Fig. 7:**
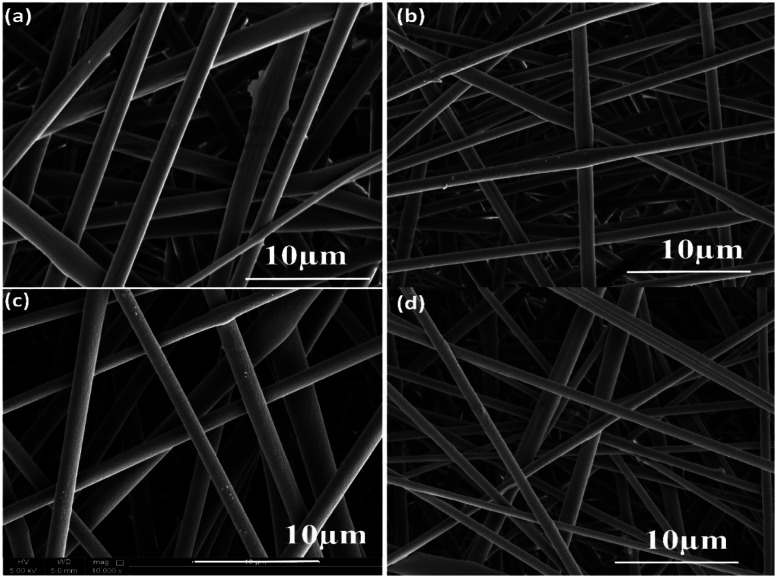
(a) SEM images of pure PLA, (b) PLA/PANI, (c) PLA/ZnO, and (d) PLA/PANI–ZnO composite fibers.

The surface morphology images of neat and composite electrospun fibers are shown in Fig. 7a–d. The PLA electrospun fibers showed a diameter of 2.5 μm, whereas the incorporation of nanoparticles decreases the fiber diameter. In the case of PLA/PANI composite, the average value obtained is 2 μm, and for PLA/ZnO, the average diameter is 1.9 μm. For PLA/PANI–ZnO composite fibers, the average fiber diameter is found to be 1.4 μm. It is found that the incorporation of PANI–ZnO composite caused the diameter of the fibers to decrease gradually from 2.5 μm to 1.4 μm. This is ascribed due to the increment in the conductivity of the solution in presence on the PANI–ZnO nanocomposite.^26^

Water contact angles are measured to confirm the surface hydrophilicity of the sample. The films hydrophilicity indicates that the sensing film absorbed water drops on the polymeric film surface. The contact angle of water drops on the sensing film surface measured by the sessile drop method. It is well known that the contact angle of a surface is less than 90°, indicates the surface is hydrophilic. As we can see in Table 1, the water contact angle for pure PLA is 78.5°, and for a PLA/PANI–ZnO composite fiber is 51.2°. The contact angle is lower for the PLA/PANI–ZnO composite fibers. This is attributed to the incorporation of PANI–ZnO is useful to increase the hydrophilicity and adsorption of water molecules on the PLA/PANI–ZnO composite fiber surface. Increased hydrophilicity has improved the water vapor absorption and settling at the surface of the nanocomposite film. An increase in the physisorption process at the sensing film surface enhances sensors sensitivity.^27^

**Table 1:** Contact angle measurements of PLA and PLA/PANI–ZnO composite fibers

Sample type	Contact angle image	Contact angle
PLA electrospun fibers	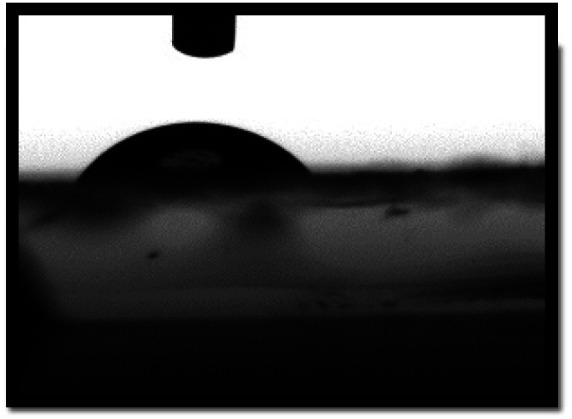	78.5°
PLA/PANI–ZnO composite fibers	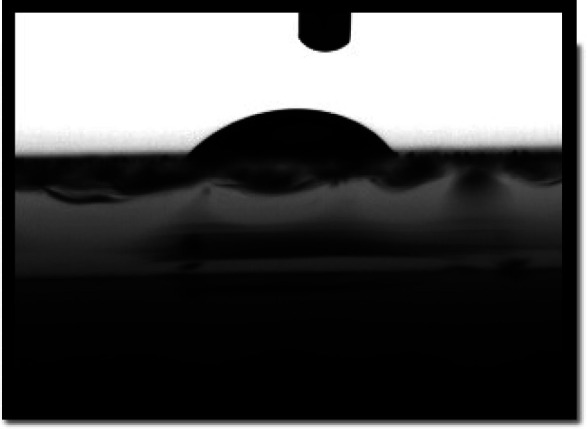	51.2°

### 3.4. Humidity sensing properties

**Fig. 8:**
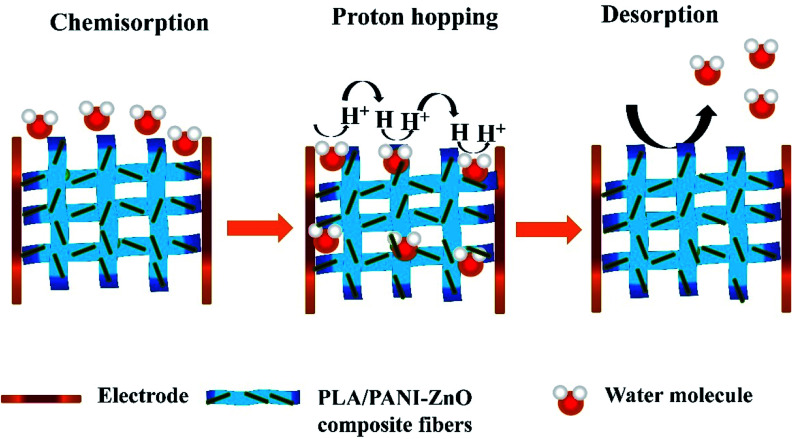
Schematic representation of the sensing process.

**Fig. 9:**
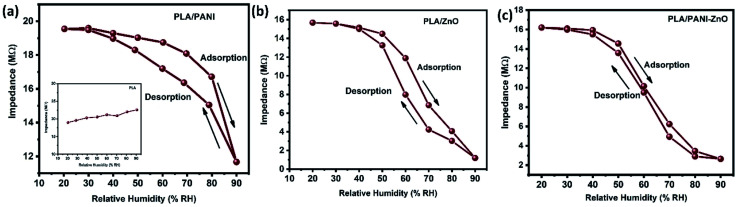
(a–c) Hysteresis characteristics of PLA/ZnO, PLA/PANI and PLA/PANI–ZnO composite fibers.

The humidity sensing properties of PLA/PANI–ZnO composites fibers is systematically investigated. The impedance response of PLA/PANI–ZnO composite based humidity sensor within the relative humidity range from 20–90% RH is shown in Fig. 9. The impedance of the PLA/PANI–ZnO composite fibers decreases with an increase in relative humidity level due to an increase in conductivity of the composite films as water vapor adsorbed on the hydrophilic surface of the composite film. At low RH, only a tiny amount of water molecules adsorb on the composite fiber surfaces. As the RH increases, more layers of water molecules are physically adsorbed. This physisorbed multilayer exhibits bulk liquid-like behavior. In this situation, (H_3_O^+^) can be readily dissociated into H_2_O and H^+^ ions and acts as significant charge carriers. At high RH condition, the free water can interpenetrate into the interlayer of PLA/PANI–ZnO composite, which reduces the rate of change of impedance due to the high dielectric constant of H_2_O.^28^ The observed highest humidity sensitivity for PLA/PANI–ZnO composite is due to its 1D fibers structure and the hydrophilic surface of the composite. The adsorption mechanism of water molecules on the PLA/PANI–ZnO composite fibers is shown in Fig. 8. The net-like structure of nanofibers makes the adsorption of water vapors on the surface of the material accessible. On the other hand, the large surface area to volume ratio of 1D nanofiber structures makes composite fiber highly sensitive.

**Fig. 10:**
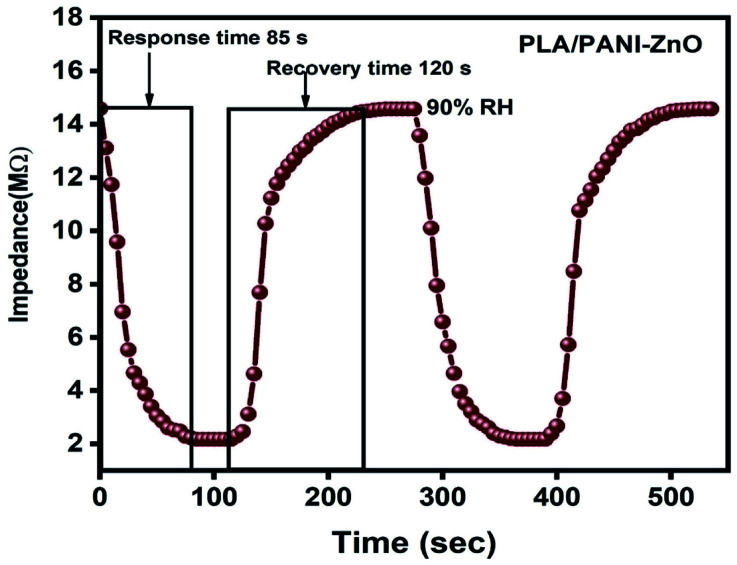
Response and recovery cycle of PLA/PANI–ZnO composite fibers based resistive humidity sensors operate at 25 °C.

Fig. 9a displays the hysteresis characteristics for PLA/PANI–ZnO composite fibers. Hysteresis is one of the critical parameters to characterize the reliability of the humidity sensor. Hysteresis is defined as the maximum difference in measured values of relative humidity during the adsorption and desorption process. The hysteresis response of PLA/PANI–ZnO composite fiber-based resistive sensor can be divided into two stages, lower humidity level (20–50% RH) and higher humidity level (60–90% RH). At a lower humidity level, the maximum calculated hysteresis at 50% RH is determined to be ∼4.2%. As the humidity level increases (60–90% RH), more water molecules adsorbed on the sensing film, and the sensors exhibit more hysteresis. The maximum calculated hysteresis at 70% RH is ∼8.9%. A smaller hysteresis loss observed for the PLA/PANI–ZnO composites. This indicates that a faster equilibrium can be reached between the adsorption and desorption process in the PLA/PANI–ZnO composites fibers. The small hysteresis effect arises due to the 1D PLA/PANI–ZnO fibers structures of the sensing film. The 1D structure of the fibers has a large surface area, which provides more sites for the adsorption of water molecules and facilitates the fast transmission of sensing molecules or ions. The response and recovery times are essential for estimating the performance of humidity sensors. Fig. 10 demonstrates the response and recovery time curves of PLA/PANI–ZnO composites based humidity sensor. The response and recovery times of PLA/PANI–ZnO composites humidity sensors are calculated to be 85 s and 120 s. It indicates that the PLA/PANI–ZnO sensor exhibited the best response and recovery behavior. The sensor with PLA/PANI–ZnO has been selected for the repeatability test because of its highest sensitivity and it shows that the same response curve during the cyclic test, which indicates its excellent repeatability.

## 4. Conclusion

In the present work, PANI–ZnO nanocomposite is synthesized by the hydrothermal method and *in situ* polymerization method. The electrospinning technique is used to prepares PLA/PANI–ZnO composite fibers. The prepared PANI–ZnO nanocomposite has a mixed morphology of rods and nanoparticles observed in SEM and TEM micrographs. The average fiber diameter of the PLA/PANI–ZnO composite fibers has decreased with the addition of PANI–ZnO nanofillers. DSC results demonstrate that the addition of nanoparticles increases the thermal properties of the PLA matrix. The humidity sensing of the PLA/PANI–ZnO composite fibers are investigated in the RH range 20–90% at room temperature. The experimental results showed that the composite fibers-based sensor exhibited a response time of 85 s and recovery time 120 s. These results indicated that 1D fiber structures improve the performance of humidity sensors.

## Data availability

The data that support the findings of this study are available from the corresponding author upon reasonable request.

## Author contributions

All authors contributed equally.

## Conflicts of interest

The authors declare that there is no conflict of interest.

## Supplementary Material
